# Cyberpsychiatry versus COVID-19: using video consultation to improve clinical care in an in-patient psychiatric unit

**DOI:** 10.1192/bji.2021.19

**Published:** 2021-08

**Authors:** Sarah Moslehi, Dominic Aubrey-Jones, Melanie Knowles, Janet Obeney-Williams, Senem Leveson, Golnar Aref-Adib

**Affiliations:** Camden & Islington NHS Foundation Trust, London, UK, email: sarah.moslehi@nhs.net

**Keywords:** COVID-19, pandemic, telehealth, telepsychiatry, cyberpsychiatry

## Abstract

The COVID-19 pandemic has resulted in unique challenges for in-patients across the National Health Service as visitors, both family and friends, are prevented from visiting patients owing to infection prevention and control measures. The Attend Anywhere platform was used as the basis of a quality improvement project to mitigate the detrimental effects of reduced social contact for patients. The use of video conferencing led to increased subjective satisfaction for both patients and healthcare professionals, thereby providing further evidence of the benefit that this emerging technology has on healthcare delivery.

## Video calling on a COVID-19 ward

In March 2020, our acute admissions ward in North London became our trust's allocated isolation ward for psychiatric patients with confirmed or suspected COVID-19 infection. The pandemic created a variety of unprecedented challenges across the trust, one of these being that visitors, including healthcare professionals from community teams, were no longer permitted access to the in-patient wards.^1^

Many patients on the wards no longer had regular contact with their friends and family. This created anxiety for carers and relatives, and they reported dissatisfaction with telephone call updates from ward staff. In turn, the patients reported feeling increasingly disconnected from their families.

The Attend Anywhere software provided a solution to these types of problem. A video consulting platform that is both confidential and recommended for use within the National Health Service (NHS), Attend Anywhere allowed patients to have face-to-face video conferences with family and friends, as well as allowing healthcare professionals to attend patient meetings remotely.^2^ The use of Attend Anywhere removed some of the unprecedented barriers created by the COVID-19 pandemic, and we found that both patient and next of kin satisfaction improved.

## Video consultation software

**Fig. 1:**
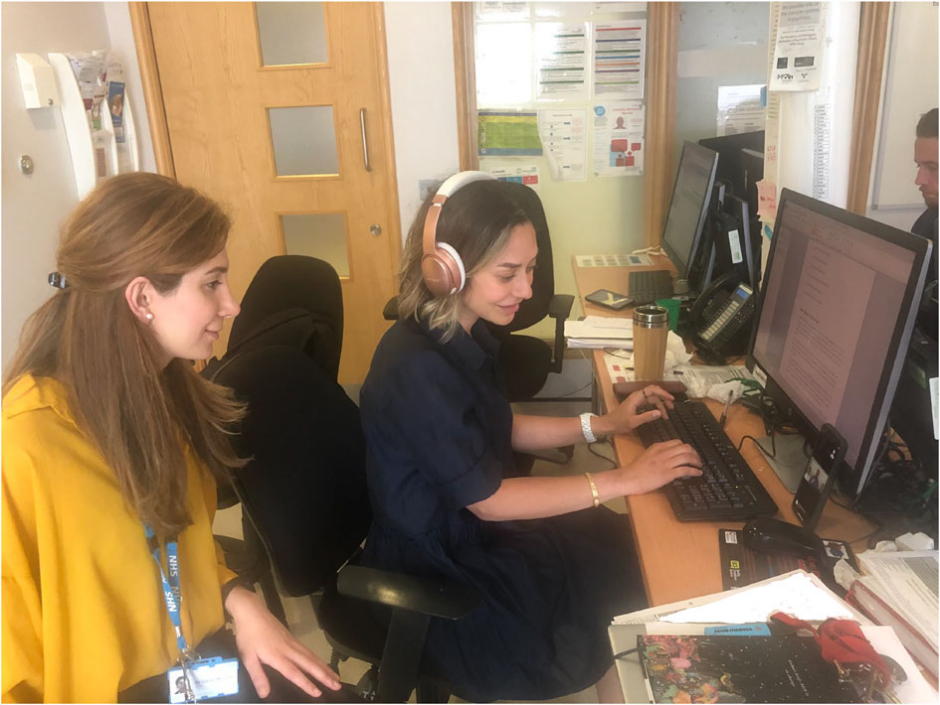
Dr Moslehi and Dr Leveson using Attend Anywhere.

The software was easily used on the ward. Attend Anywhere accounts were created for staff with their professional email address. Family members and healthcare professionals were given an URL link to the healthcare professional's Attend Anywhere account and could join the video consultation. The URL link was given either over the phone or via email. The attendees used the URL link and entered a virtual waiting area until the healthcare professional accepted the video call. (See Fig. 1, where doctors on Sapphire ward are using the Attend Anywhere software during a patient consultation).

**Patient experience 1**

Patient X, who was admitted with a relapse of paranoid schizophrenia, came to our ward after testing positive for COVID-19 and required a mandatory 7 day period of isolation. The patient's next of kin complained about not getting sufficient updates about the patient and reported that they were anxious about his mental and physical health.

In case of Patient X, the ward team facilitated video consultations on a regular basis and enabled the patient's next of kin to see and talk to him and to meet the team. This facilitated a relationship between the mental health professionals and patient X's family, allowing them to contribute in a more meaningful way to their son's care. Attend Anywhere video consultations reduced the anxiety of the family members, and this was reflected in an improvement in the carer satisfaction scores received by the ward.

*‘I have not been able to visit my son for weeks. Thank you for the video calls. I am so glad that I can see him now’ – Patient X's mother*

## Expanding video calling across the trust

The Attend Anywhere video consultation platform was not widely used by healthcare professionals in our NHS trust before the COVID-19 pandemic. The necessary safety measures in response to the pandemic led to restrictions in the level of contact that healthcare professionals had with relatives and colleagues. Our team therefore embarked on a quality improvement project to promote video consultation via Attend Anywhere as a means of enhancing patient care.^3^

Information was provided to other teams in the form of brief in-person teaching sessions and written leaflets. A list of interested staff members was compiled, and these individuals were proactively contacted by the trust lead to set up new accounts. This hands-on approach continued with email prompts and face-to-face support for technology or IT difficulties.

‘*Video consultation is a very useful tool for health care professionals to contact patients’ next of kin.’ – Doctor*

Attend Anywhere usage data were requested from the trust lead every 2 weeks to evaluate the increase in its uptake and ongoing use.

**Patient experience 2**

Another individual, Patient Y, was suffering from a first episode of psychosis and had recently moved to the UK from another continent to attend university. He did not have any family members in the UK. His relatives could not enter the UK owing to the pandemic. They were also not familiar with mental healthcare services in the UK, which further heightened their anxieties.

Attend Anywhere was used to invite Patient Y's parents to his care planning meetings, enabling them to participate in his care and allowing a dialogue between them, the patient and the healthcare team. Patient Y's parents were able to see the faces of the doctors and nurses caring for their son, and to visualise the ward environment from another continent. This reduced some of their uncertainty and confusion surrounding his treatment and greatly alleviated their anxiety about his care.

## Challenges and solutions

One of the greatest obstacles to Attend Anywhere usage was poor internet connection on the wards and lack of staff access to devices such as laptops and iPads. Many staff members reported that they had to use their own smartphones. Other challenges included difficulties of relatives or carers in using the technology, or their lack of access to a suitable device.

Participants in the quality improvement project raised the issue of poor internet connection and identified it as a major factor in prevention of video consultation. Therefore, the problem of poor internet connectivity was reported to the trust's IT department by the team and was subsequently solved. The quality improvement team also spoke to ward managers and asked whether they could order more devices for staff to use for video consultations.

## Positive factors leading to an increased uptake

Factors that enabled implementation of Attend Anywhere across the trust were the positive feedback and interest of staff members, the preference voiced by family members for its use in place of telephone calls, and the timesaving nature of video consultation. The collective experience throughout our hospitals was that Attend Anywhere facilitated the containment of the COVID-19 virus, as more patients were willing to self-isolate when they had a means of closely communicating with their friends and family.

## Scope for international use of telepsychiatry

The use of telepsychiatry during the COVID-19 pandemic on our ward has highlighted areas in which its use could be further expanded internationally. Many rural areas with a low population density are unable to afford or justify secondary care psychiatric input;^4^ these areas could be linked to psychiatry clinics in regional centres using video consultation platforms such as this one. Potential barriers to this include limited access to technology in rural areas of less economically developed countries. Given the significant difficulties with the internet connection in our hospital, it is likely that this would be a potential barrier to the uptake of telepsychiatry worldwide. Other potential barriers to an effective implementation of telepsychiatry nationally as well as internationally include insufficient resources and lack of familiarity with the required technology.^4^

The use of telehealth and its effectiveness are not limited to the pandemic. There is room for further development to enable healthcare professionals from two or more remote sites, nationally as well as internationally, to connect and contribute to patient care.^5^

Adaption to telepsychiatry in general is reliant on a variety of factors. The teleconsultation should be simple, familiar and patient-centric, as explained by Attend Anywhere founder Chris Ryan in an article about telehealth.^5^

## Conclusion

Video consultation technology is becoming widely used in medicine, and the COVID-19 pandemic has highlighted the benefits it can bring to healthcare professionals, patients and their families.

**Fig. 2:**
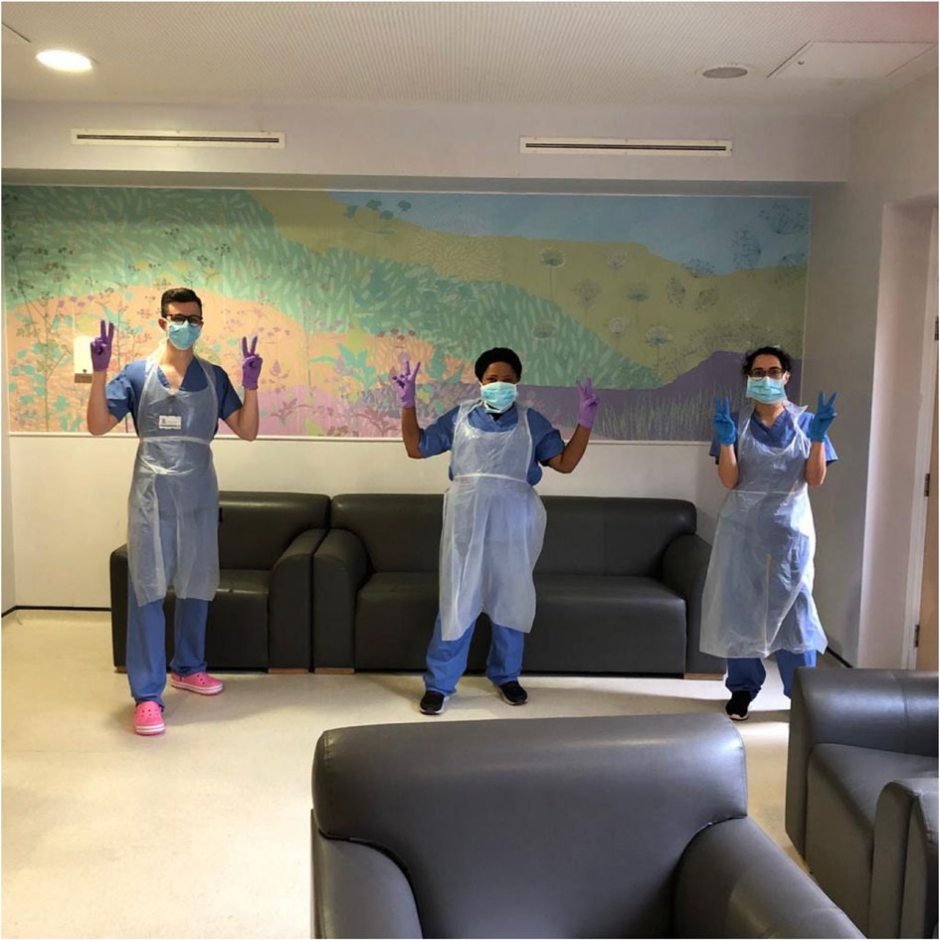
Dr Aubrey-Jones, Esther (ward manager) and Dr Aref-Adib wearing PPE. All pictured individuals have given consent for their picture to be used in this paper.

Software such as Attend Anywhere allows flexible contact with relatives and staff members from other teams, who may be unable to attend meetings in person for a variety of reasons – one reason being the necessity of personal protective equipment (PPE) usage for all staff, even those from the same facility. (Fig. 2 shows three ward staff wearing PPE).

The feedback from our patients and their friends and family was that they felt less isolated and anxious as a result of access to Attend Anywhere, while staff reported that, notwithstanding various technological challenges, it was a helpful means of remaining involved in the in-patient care of their patients during lockdown.

Its use nationally is likely to continue over the coming months, as social distancing measures remain in place, and there is a clear wider role on a global scale for remote video consulting in secondary care psychiatry.

Our unit will also continue to use video consultation beyond the pandemic, as it is an efficient way to provide patient care and attend meetings remotely.

## Supplementary material

For supplementary material accompanying this paper visit https://doi.org/10.1192/bji.2021.19. click here to view supplementary material
